# *In vitro* Culture of Naïve Human Bone Marrow Mesenchymal Stem Cells: A Stemness Based Approach

**DOI:** 10.3389/fcell.2017.00069

**Published:** 2017-08-23

**Authors:** Bidisha Pal, Bikul Das

**Affiliations:** ^1^Department of Immunology and Infectious Diseases, The Forsyth Institute Cambridge, MA, United States; ^2^Department of Stem Cell Biology, KaviKrishna Laboratory, Guwahati Biotech Park, Indian Institute of Technology Guwahati, India

**Keywords:** stem-cell based regenerative therapeutics, self-renewal, stemness, naïve MSCs and *in vitro* primed MSCs, CD271+ BM-MSCs, Altruistic stem cells (ASCs)

## Abstract

Human bone marrow derived mesenchymal stem cells (BM-MSCs) resides in their niches in close proximity to hematopoietic stem cells (HSCs). These naïve MSCs have tremendous potential in regenerative therapeutics, and may also be exploited by cancer and infectious disease agents. Hence, it is important to study the physiological and pathological roles of naïve MSC. However, our knowledge of naïve MSCs is limited by lack of appropriate isolation and *in vitro* culture methods. Established culture methods use serum rich media, and serial passaging for retrospective isolation of MSCs. These primed MSCs may not reflect the true physiological and pathological roles of naive MSCs (Figure 1). Therefore, there is a strong need for direct isolation and *in vitro* culture of naïve MSCs to study their stemness (self-renewal and undifferentiated state) and developmental ontogeny. We have taken a niche-based approach on stemness to better maintain naïve MSCs *in vitro*. In this approach, stemness is broadly divided as niche dependent (extrinsic), niche independent (intrinsic) and niche modulatory (altruistic or competitive). Using this approach, we were able to maintain naïve CD271+/CD133+ BM-MSCs for 2 weeks. Furthermore, this *in vitro* culture system helped us to identify naïve MSCs as a protective niche site for Mycobacterium tuberculosis, the causative organism of pulmonary tuberculosis. In this review, we discuss the *in vitro* culture of primed vs. naïve human BM derived MSCs with a special focus on how a stemness based approach could facilitate the study of naïve BM-MSCs.

## Introduction

Bone marrow (BM) stem cell niche is the home to the quiescent hematopoietic stem cells (HSCs). Until stimulated by injured-tissue derived signals for regenerative purposes, HSCs remain in their quiescent state perpetuating for a lifetime capacity to self-renew. The niche also contains mesenchymal stem cell (MSC) population residing in close proximity to hematopoietic stem cell (HSC) (Bara et al., 2014). HSCs differentiate to erythrocytes, thrombocytes, and leukocytes, whereas MSCs gives rise to cartilage, fat and bone cells. In recent decades, there has been a tremendous interest to isolate and culture these BM-MSCs due to their therapeutic potential in stem cells based regenerative medicine (Prockop, 2017). For experimental and therapeutic purposes, freshly obtained BM mononuclear cells are subjected to culture in plastic adherent dishes, thereby giving rise to a heterogeneous population of cells, known as mesenchymal stromal or MSCs. These cells are further injected to mice or human for evaluating their regenerative capacity. Interestingly, several clinical trials have been conducted since 1995 that confirms the sustained interest on this cell type. However, this interest is mainly based on the speculation that similar to HSCs; MSCs could be another quiescent stem cell population that may self-renew and home to injured tissues for regeneration. However, unlike HSCs, the stem cell characteristics of MSCs are not yet confirmed. Part of the reason is the confusion that prevails in the isolation and culture of a homogeneous population of naïve BM-MSCs. In this review, we intend to discuss the challenges of *in vitro* culture expansion of primed (*in vitro* culture expanded) vs. naïve BM-MSCs and address the growing interest to take a stemness-based approach to study naïve BM-MSCs.

**Figure 1:**
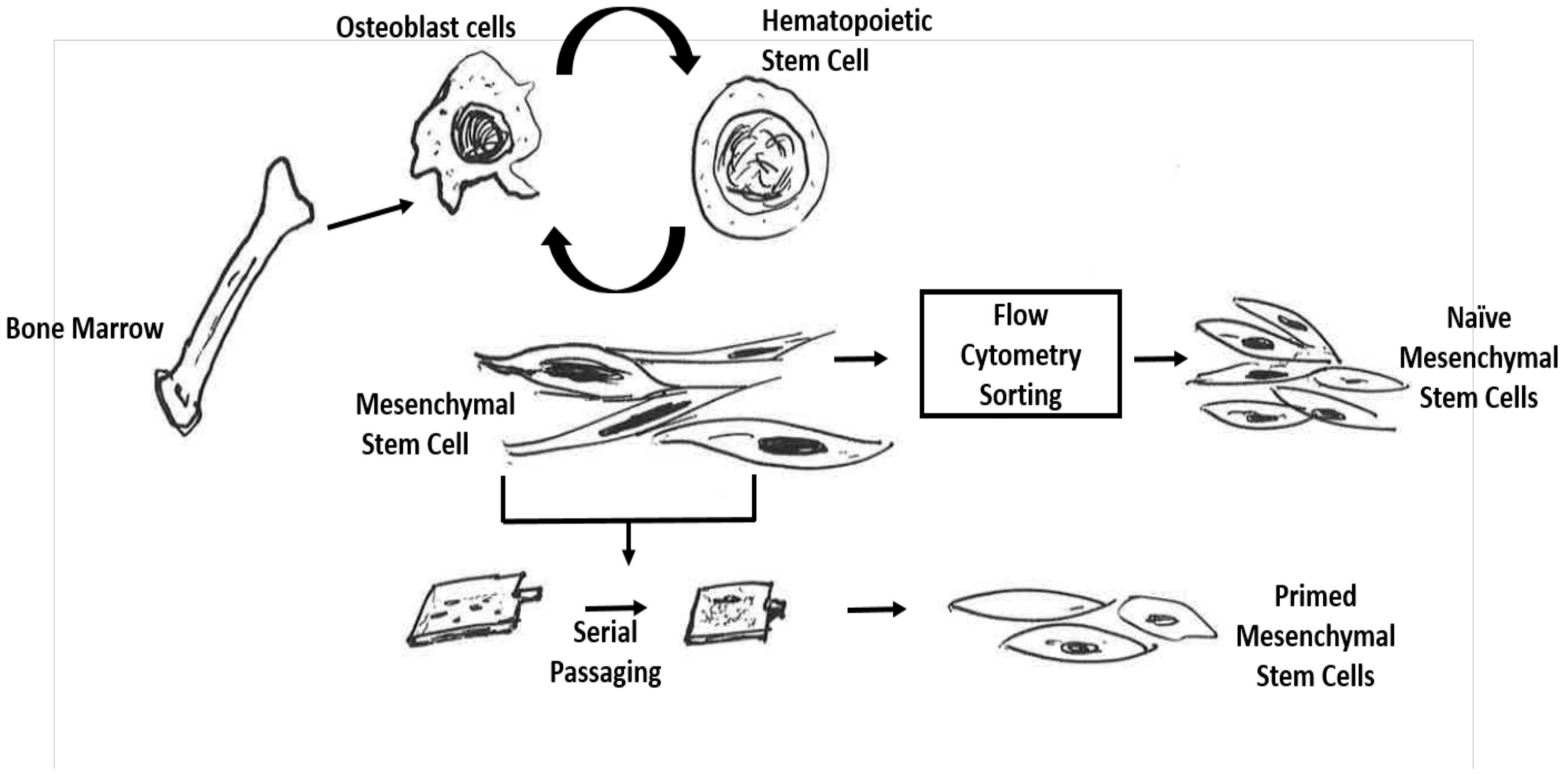
Schematic representation to demonstrate difference between naïve and primed bone marrow MSCs. Naïve mesenchymal stem cells are obtained by first isolating the bone-marrow mononuclear cells and then subjecting them to flow cytometry sorting based on promising cell surface markers for naïve MSCs such as CD271. In contrast, the *in vitro* primed mesenchymal stem cells are procured by initially obtaining the BM-MNCs cell population and then directly subjecting these cells to *in vitro* serial passaging in high serum containing media.

Conventionally, for the *in vitro* expansion of MSCs, BM mononuclear cells are cultured in plastic adherent dishes under high serum conditions. Following 2–3 passages, the adherent cells are collected and found to be highly enriched in MSCs (Figure 1; Friedenstein et al., 1987; Kuznetsov et al., 1997; Dolley-Sonneville et al., 2013). These *in vitro* culture expanded MSCs could be termed as primed MSCs as these cells are primed or adapted to its microenvironment during the *in vitro* expansion in serum rich culture media. These primed MSCs exhibit multipotency (Pittenger et al., 1999), secretion of growth factors, and anti-inflammatory molecules, (Iyer and Rojas, 2008), (Uccelli et al., 2008) that may promote cell survival, angiogenesis and immune modulation (Haynesworth et al., 1996; Caplan and Bruder, 2001; Chen et al., 2008). Interestingly, several studies indicate that these cells possess the heterogeneous ability to differentiate into nerve cells (Rooney et al., 2009), hepatic cells (Lee et al., 2004) and cardiac cells (Kawada et al., 2004) suggesting their immense potential to repair and heal injured tissues upon transplantation to the host.

Although above mentioned functional properties of primed MSCs may appear fascinating and clinically relevant, whether these properties are acquired or selected during prolonged retrospective expansion in serum rich media, is an ongoing controversy (Pacini and Petrini, 2017). Importantly, the physiological relevance of these properties of primed MSCs is not yet known as the naïve counterpart of these primed MSCs are not yet identified. For example, primed MSCs were found to be immunosuppressive in nature, possibly due to their low expression of antigen presenting molecules and lack of expression of Major Histocompatibility class (MHC) II molecules and B7 co-stimulatory molecules for MHC class I (Siegel et al., 2009). Indeed, these properties make primed MSCs a promising candidate for regenerative therapeutics including cell therapy for graft-vs.-host disease (GVHD) (Keating, 2012) and autoimmune disorders (Rafei et al., 2009). However, the physiological relevance of MSC mediated immunosuppression is not yet clear. It could be assumed that avoiding immune detection by MSCs may provide niche support to HSCs against immune reaction. This assumption is supported by studies showing that co-injection of MSCs with HSCs increased the immune tolerance of transplanted HSCs (Chung et al., 2004; Li and Wu, 2011; Vanikar et al., 2011). However, this assumption is not yet confirmed in naïve MSCs. In fact, the retrospective isolation approach of MSCs has further added substantial ambiguities in the identification, isolation, expansion and characterization of naïve -MSCs (Figure 1; da Silva Meirelles et al., 2008). Importantly, unlike HSC research that attracted developmental biologists to study the ontogeny of these cells, MSC research has not seen such enthusiasm. The reasons are complex, but possibly due to the inability to obtain a homogeneous naïve MSC population that could be reproducibly isolated and maintained *in vitro* in laboratories across the world.

**Figure 2:**
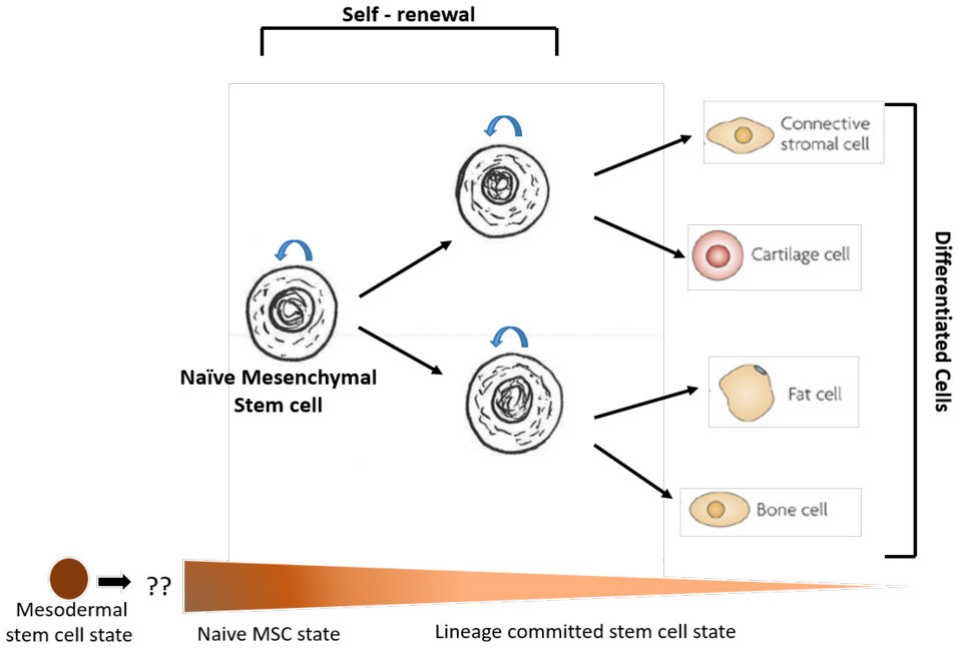
A schematic representation of the self-renewing compartment of bone marrow (BM) naïve mesenchymal stem cell (MSC) and its commitment to mesenchymal lineages. The naïve MSCs are defined as the bone marrow MSCs that participate in the BM niche of hematopoietic stem cells (HSCs). The MSCs present in other tissues including adipose tissues does not comply with the definition of BM derived naïve MSCs. The spectrum figure below denotes potential developmental ontogeny of MSC stemness states comparable to the spectrum of cell state according to the Waddington's epigenetic landscape (Das, 2014). The naïve MSC state is probably derived from the lateral plate mesodermal cells or mesodermal stem cells, although, this speculation needs to be confirmed by rigorous research.

BM-MSCs constitute a rare fraction (< 0.001%) of the BM- mononuclear cells (Friedenstein et al., 1987; Pittenger et al., 1999), which makes the isolation and expansion of naïve MSCs *in vitro* quite challenging. Fortunately, several cell surface markers have been identified, such as Stro-1, CD271, CD146 and CD133 to enrich the naïve BM-MSCs cell population. *In vivo* studies indicated that CD271+ BM-MSCs resides in the hypoxic niche of BM, whereas CD146+ MSCs reside in the perivascular niche (Tormin et al., 2011). The specialized niche in BM may also support the stemness (self-renewal and undifferentiated state) of MSCs and HSCs. Based on these advances here we shall define naïve MSCs as the flow cytometry sorted BM-MSCs that resides in the HSC niche of BM (Figures 1, 2). As per this definition, CD271 cell surface marker could be a promising candidate to isolate naive BM-MSCs. Indeed, we found that most of the CFU-Fs for the lineage-/ CD45- BM cells were enriched in CD271+ cell population (Das et al., 2013; Table 1). This observation has been independently confirmed by others investigators (Quirici et al., 2002; Buhring et al., 2007; Tormin et al., 2011).

**Table 1:** Serum-free culture system maintains the naïve BM-MSC phenotype of CD271+ /CD133+ BM cells^*^.

**MSC phenotype assessment**	**CD271+/CD133+serum free**	**CD271+/CD133+20% FBS**
CD271	92.7 ± 3.6	11.0 ± 4.3
CD133	73.0 ± 8.5	8.0 ± 2.1
CD45	24.0 ± 9.7	9.3 ± 2.1
CD105	47.7 ± 12.2	84.4 ± 21.8
CD73	56.3 ± 8.7	75.0 ± 14.4
CD90	21.0 ± 6.5	73.5 ± 15.2
VEGFR2	2.0 ± 1.2	1.2 ± 0.4
ABCG2	24.0 ± 19.6	1.1 ± 0.4
HIF-2α	20.0 ± 17.2	2.1 ± 1.4
Oct-4	-	+
CFU-F/10^6^ cells^**^	4349 ± 1936	3910 ± 1993

(*) *CD271+/CD133+ BM cells were cultured for 2 weeks with or without serum plus TPO, Flt3 ligand and SCF and then subjected to flow cytometry analysis. Note that about 92% of cells remained positive for CD271 in the serum-free culture system whereas only 11% cells remain CD271+ when grown in the presence of serum. The CD271+/CD45- BM-MSCs isolated from the 2-weeks culture exhibited MSCs phenotype including differentiation potential to three lineages. CFU-F of the CD271+/CD45- BM-MSCs are given in the table (details are given in Das et al., 2013). Oct-4 values were obtained by QPCR*.

** *CFU-F was obtained by first immunomagnetic sorting of CD271+/CD45- cells from the 2 weeks old culture of CD271+/CD133+ BM- cells, and then subjecting to CFU-F assay. 34% of the BM cells were CD271+/CD45- BM-MSCs. This table was published in Das et al. (2013)*.

The current approach is to culture the naïve MSCs cells in high serum conditions or serum free media with defined growth factors. However, this approach has not been successful as the addition of serum has been shown to induce differentiation of naïve BM-MSCs (Das et al., 2013; Table 2).

**Table 2:** Summary of the approaches to culture naïve BM-MSCs in the serum free culture with growth factor supplements.

**Marker used**	**Approach to culture naïve BM-MSC**	**Fate of naïve MSCs**	**References**
Stro-1/ CD45-	Stro-1/CD45- BM-MSCs cells were immunomagnetically sorted and cultured *in vitro* using serum free alpha-MEM media formulation (BSA, insulin, transferrin and low-density lipoprotein) with various growth factors and or L-ascorbate. The Stro-1+ cells also expressed CD271, PDGF-R and EGF-R.	CFU-F was increased in the presence of L-ascorbate, PDGF and EGF. The maintenance of Stro-1 and or CD271 expression during *in vitro* culture was not studied.	Gronthos and Simmons, 1995
Ficoll separated human BM cells	BM mononuclear cells were cultured and maintained in a serum-free media containing IMDM supplemented with 1% BSA, 5 ug/ml of human insulin, 100 ug/ml of human transferrin, 10 ug/ml of low-density lipoprotein, 10^∧^-4 M beta-mercaptoethanol and growth factors (SCF, and IL-3).	CD123+/CD45- mesenchymal progenitor cells with osteogenic differentiation ability could be expanded for 3weeks.	Baksh et al., 2003
Ficoll separated human BM cells.	Stirred suspension culture in Stem Span media, (StemCell Technologies, Vancouver, BC) with SCF and IL-3.	Concomitant growth of both CD45+ and CD45- cells was observed. CD123+/CD45- cells were maintained for 3 weeks and showed 3-fold increase in number.	Baksh et al., 2005
CD271+/ CD56+, and CD271+/ CD56-	CD271+/CD56+ or CD271+/CD56- cells were grown in serum free media with FGF-2 supplement. Duration of *in vitro* culture is not mentioned.	CD271+ expression was lost, whereas the expression of CD166 and CD318 were increased.	Battula et al., 2009
CD133, CD271, CD45	Flow cytometry sorted population of CD133+/CD271+/CD45- BM-MSCs were cultured *in vitro* in serum rich media.	Loss of CD133 and CD271 marker expression. Also, marked reduction in CFU-F formation as compared to freshly obtained CD133+/CD271+/CD45- cells.	Bakondi et al., 2009
CD146, CD271	CD146+/CD271+ BM-MSCs were maintained in serum rich media.	CD271 expression in BM-MSCs was decreased during the *in vitro* culture system using serum rich media.	Tormin et al., 2011
CD133+CD271+ BM cells	CD133+/CD271+ cells that contain both HSCs and MSCs were cultured in serum free StemSpan media with growth factors SCF, TPO, and Flt3. IL-3 was not added to avoid the expansion of osteogenic progenitor as observed by Baksh et al. (2003).	92% of CD271+ BM-MSCs maintained stemness for 2 weeks, and showed 2-fold expansion.	Das et al., 2013
CD34+/ CD45+ and CD271+/CD45-	CD271+ BM-MSCs were co-cultured with human umbilical cord blood CD34+ cells in serum free media with SCF, TPO, and Flt3. *In vitro* culture was done for a week.	CD271+ cell surface marker expression was preserved for 1 week.	Li et al., 2014

**Figure 3:**
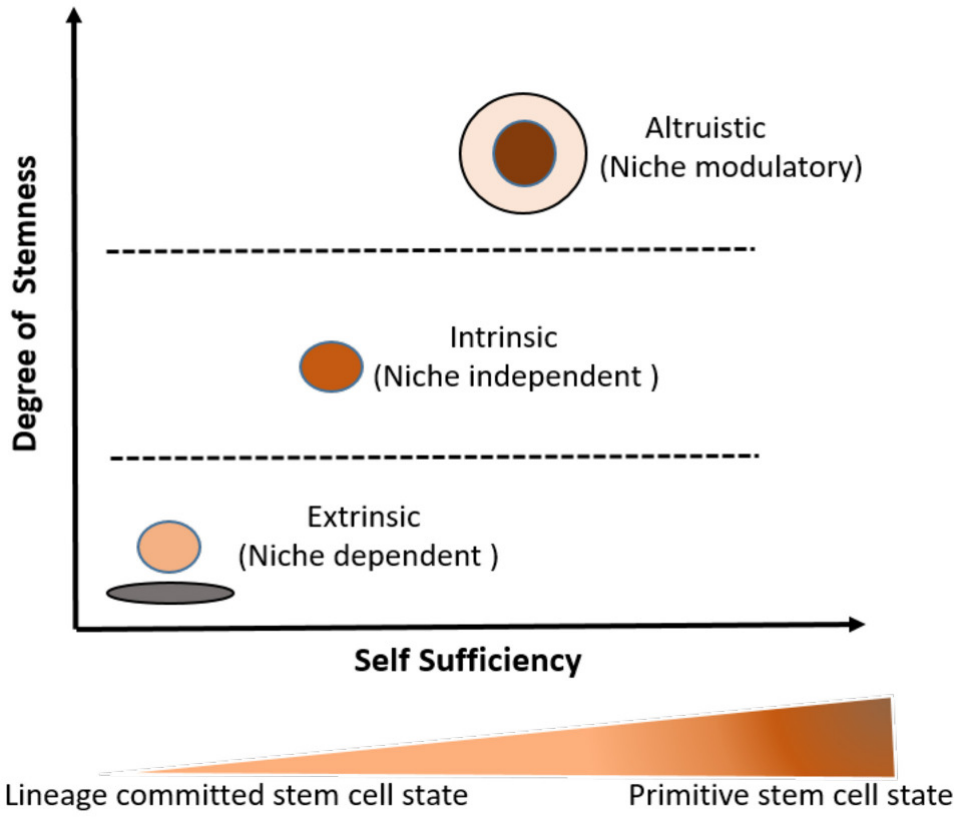
Three broad types of stemness (self-renewal and undifferentiated state) as a relational attribute to stem cell niche and developmental ontogeny of the stem cell undifferentiated state. The idea of the degree of stemness incorporates both stem cell niche and developmental/hierarchical ontogeny of a given stem cell state. Higher the degree of intrinsic stemness, higher is the level of self-sufficiency and the hierarchical position of the stem cell state in the developmental ontogeny of stem cells. In this model, primitive stem cell state such as naïve ES cells exhibit higher degree of intrinsic stemness and are more self-sufficient than lineage committed stem cells such as CD34+ HSCs. The detail discussions on the idea about the degree of stemness and self-sufficiency (including niche modulatory altruistic stem cell state) is given in two book chapters (Das et al., 2009; Das, 2014).

An alternative approach could be taking a stemness-based approach. Stemness is the self-renewal and undifferentiated property. Arai and Suda took a niche-based approach to define stemness as weak and strong-niche stemness (Arai and Suda, 2008). We have previously discussed this approach in the context of stem cells and cancer (Das et al., 2009; Das, 2014). In this approach, stemness is described broadly as a gradient of stem cell self-renewal and undifferentiated state (Das, 2014), where one end describes the niche dependent (extrinsic) stemness while the other end demonstrates the niche independent (intrinsic) stemness. In this spectrum, higher level of intrinsic stemness leads to self-sufficiency or niche independency, i.e., ability to maintain stemness without serum or any growth factor support (Figure 3). Our lab characterized this unique feature of niche independent stemness in ES cells (Das et al., 2012).

Naïve MSCs being part of the HSC niche may exhibit extrinsic stemness, and therefore, co-culturing these MSCs with HSCs might facilitate to maintain the stemness state of naïve MSCs. Indeed, we found that when CD271+/CD133+ MSCs were co-cultured with CD133+ hematopoietic cells, the CD271+ MSCs could maintain their naïve stemness state (Das et al., 2013). We then used this stemness-based *in vitro* assay to study the pathogenic role of naïve MSCs in cancer and infectious diseases. We reported that pathogen could exploit the stemness of MSC for immune evasion (Das et al., 2013; Garhyan et al., 2015; Lopes et al., 2016). Interestingly, we also found that cancer cells may modulate the stemness of naïve BM-MSCs to favor tumor growth (Talukdar et al., 2016; Talukdar et al., under review). These studies further emphasize that naïve MSCs and their stemness may be of importance in the pathogenesis of infectious diseases and cancer. Furthermore, using the *in vitro* assay, we found that cancer stem cells could switch the stemness of naïve CD271+ BM-MSCs from extrinsic to altruistic stemness state (Talukdar et al., under review).

Altruistic stemness (Table 3) is the special feature of those stem cells that exhibit altruistic behavior. Altruistic behavior is well reported in bacteria as a defense strategy (Youk and van Oudenaarden, 2010). We recently described a similar altruistic behavior in human embryonic stem cells exposed to oxidative stress (Das et al., 2012). Stem cell altruism could be viewed in opposition to stem cell competition. Thus, stem cell competition is a fitness-sensing mechanism that eliminates weak neighbors in the niche (Di Gregorio et al., 2016). In contrast, stem cell altruism could be defined as a fitness-defense mechanism that protects weak neighbors in their niche. The mechanisms of stem cell competition and altruism may play important roles in the physiological, regenerative and pathological roles of naïve MSCs, and therefore, it is important to an optimal *in vitro* assay to study altruistic and competitive behaviors of naive MSCs. This need could be partially fulfilled by taking a stemness-based approach to culture naïve MSCs.

**Table 3:** Glossary of Terms.

**Term**	**Definition**
Naïve MSCs	MSCs that form the part of HSC niche in BM.
Primed MSCs	MSCs that are obtained after culturing and serial passaging of BM-MNC in serum rich media.
Stemness	Self-renewing and undifferentiated state of stem cells.
Extrinsic or niche dependent stemness	Stem cells that rely on external factors secreted by niche cells to maintain their stemness.
Intrinsic or niche independent stemness	Stem cells that secrete their own soluble factors to maintain stemness. However, these cells require certain degree of niche support to maintain stemness.
Self sufficiency	Self – sufficiency is an extreme form of intrinsic stemness where stem cells exhibit complete independency to niche while maintaining their stemness.
Altruistic or niche modulatory stemness	Type of stemness that emerges in stem cells during oxidative stress. Altruistic stemness is characterized by high level of HIF-2alhpa, glutathione and low level of p53, as well as self-sufficiency. It is a transient state of stemness, as the cells eventually differentiate and or undergo apoptosis/senescence. Importantly, the cells having altruistic stemness secrete glutathione and other factors that modulate the niche to favor the survival/growth of resident cells (Das et al., 2012; Das, 2014).
Altruistic stem cells (ASCs)	Stem cells with extrinsic or intrinsic stemness that reprogram to altruistic stemness during stress. These cells exhibit stem cell altruism.
Stem cell altruism	Stem cell altruism is a fitness-defending mechanism that defends/protects neighboring stem cells with low fitness. Altruistic stem cells acquire a “super-fit” state by down regulating p53, and secrete factors to increase the fitness of its own members, and then self-sacrifice by undergoing spontaneous apoptosis/differentiation (Das et al., 2012). The mechanism could play important role in tissue regeneration, as well as genesis of cancer (Das, 2014; Di Gregorio et al., 2016). Stem cell altruism could be better understood in context of stem cell competition.
Stem cell competition	In opposed to stem cell altruism, stem cell competition is a cell fitness-sensing mechanism that eliminates neighboring cells with low fitness. The mechanism is highly conserved in evolution and most likely to play important roles in tissue homeostasis and stem cell maintenance in their niche (Di Gregorio et al., 2016).

Thus, studying naïve MSCs would not only help us to develop better regenerative therapies but also would provide deep insights about the role of MSCs in the pathogenesis of cancer and infectious diseases. Therefore, in this review, we will discuss the limitations of primed MSCs in understanding the physiological properties of MSCs in general, and thereby the need to focus on isolating and characterizing the naive state of MSCs using a stemness-based approach. Then, we will discuss how this stemness based approach of *in vitro* culturing of naïve MSCs could help us to understand the regulation of stemness by immune cells, cancer and pathogens.

## Limitations of *In vitro* MSCs, primed MSCs or mesenchymal stromal cells

The existence of a non-hematopoietic component in bone marrow was first suggested by Cohnheim (1867). Subsequently, Friedenstein and coworkers isolated the adherent colony-forming fibroblast- like cells (CFU-Fs) from whole bone marrow cell suspensions (Friedenstein et al., 1970, 1974a,b) which initiated the research on multipotent MSCs. Generally, the *in vitro* culture expanded or primed MSCs were isolated by culturing the bone marrow mononuclear cells suspension (MNCs) in 10% fetal bovine serum (FBS), new-born calf serum (NBCS) or fetal calf serum (FCS) at 37°C and 5% CO_2_ in ambient O_2_ concentration for a week (Sekiya et al., 2002; Beyer Nardi and da Silva Meirelles, 2006). Subsequently, the non-adherent cells were discarded to favor the selective expansion of the plastic adherent MSCs under high serum condition. However, the presence of other abundant cell types in the cell suspension adhering to the tissue-culture flask might hinder the isolation of the pure population of MSCs. Hence, it was recommended to culture these cells for a prolonged period to remove the contaminating cells from the heterogeneous population. However, during this process of selectively isolating the adhering MSCs from the mixed population, the possibility of acquiring new genotypic changes or reprograming of the non-MSCs cell type to MSCs, and or phenotypic alteration of the growing MSCs could not be ruled out (da Silva Meirelles et al., 2008; Trombi et al., 2009).

There are several issues of culturing primed MSCs that hinders our progress to better understand naïve MSC stemness. First, the results of *In vitro* expansion of BM-MSCs has been highly heterogeneous (da Silva Meirelles et al., 2008; Pacini and Petrini, 2017). The source of heterogeneity could be complex factors, including culture conditions and source of serum, or media preparation. Indeed, some studies demonstrated the negative influence of microenvironmental factors, such as ambient oxygen concentration (Fehrer et al., 2007; Estrada et al., 2012) and serum content (Meuleman et al., 2006) on the growth of BM-MSCs. Additionally, to efficiently culture primed MSCs, a wide variety of commercial culture media formulations, serum and other supplementation factors have been introduced but the exact composition of formulated proprietary media is fairly less understood. These *in vitro* culture-related factors might not only induce early senescence, longer population doubling time, and DNA damage (Fehrer et al., 2007; Estrada et al., 2012) but also cause poor engraftment of MSCs following transplantation to mice (Mohamadnejad et al., 2010). Taken together, these reports suggest that several complex inter- and intracellular interactive signaling systems in addition to micro-environmental factors might be controlling the growth, multiplication, and differentiation of these MSCs that could be possibly involved in priming the phenotype of these MSCs for adaption to *in vitro* culture in the *in vitro* culture system. Trombi et al proposed the “selective growth hypothesis” to explain the emergence of new stromal progenitor phenotypes in the *in vitro* culture of BM cells (Trombi et al., 2009; Cordeiro-Spinetti et al., 2014). Thus, the *in vitro* plastic adherent culture method hinders our ability to isolate and characterize naïve BM-MSCs.

Another major factor that hinders the isolation and characterization of naive MSC population is the initial heterogeneous starting culture of MSCs where only a few cells could exhibit CFU-F generation ability. To address the issue of heterogeneity and to standardize the identity of these primed MSCs, several cell surface markers that include CD105 (SH2), CD73 (SH3), CD29, CD44, CD71, CD90, CD106, CD120a, and CD124 were identified. Primed MSCs were found to be positive for these markers, and negative for CD34, CD14, and CD45 (Pittenger et al., 1999). However, only 1/3 of these MSCs exhibited true multi-lineage-potential suggesting that these cell-surface markers might not be good to enrich the pure MSCs population. Thus, heterogeneity of these primed MSCs demonstrated the shortcomings of the conventional method of MSCs isolation.

Accordingly, in 2006, the International Society for Cellular Therapy (ISCT) proposed the minimal criteria for an efficient characterization of MSCs, and renamed these cells as mesenchymal stromal cells rather than stem cells. The ISCT proposed that ≥95% of the primed MSC population must express CD105, CD73, and CD90, and only ≤2% of the population may express CD45, CD34, CD14 or CD11b, CD79a or CD19, and HLA class II (Dominici et al., 2006). Although, the standardization approach helped to minimize the ambiguities associated with the culture and study of primed MSCs, there are many issues that remained unresolved. For example, even though, ISCT states that CD34 should be used as a negative marker of MSCs, some reports suggest that the CD34 negative status is an artifact of cell culture conditions (Lin et al., 2012), (Zimmerlin et al., 2013). Importantly, the possibility that these MSCs isolated and then cultured *in vitro* would match the physiological characteristics of naïve MSCs *in vivo* or their native counterparts is less likely. Indeed, a variety of cell surface markers, such as CD44 and CD318 that are highly expressed on primed MSCs, but not in the native state of MSCs (Battula et al., 2009; Qian et al., 2012). Counter intuitively, CD271 cell surface marker is expressed in native MSCs, but loses its expression during the *in vitro* expansion in serum rich media (Quirici et al., 2002). In fact, studies have reported that MSCs might undergo phenotypic rearrangements during the *in vitro* culture conditions and thereby losing expression of some markers while also acquiring new ones (Jones et al., 2002). Thus, primed MSCs are heterogeneous in nature and they do not truly reflect or represent the properties of naïve MSCs.

## Isolation and *In vitro* culture of naïve BM-MSCs: opportunities and challenges

There has been a tremendous interest to identify the *in vivo* counterpart of primed MSCs. The current approach is to use a cell surface marker, perform *ex vivo* isolation of MSCs, and then maintain these cells *in vitro* for therapeutic purposes (Bianco et al., 2008). However, these efforts have been failed so far to bring clarity to the prevailing confusion about the *in vivo* identity of naïve MSCs (Montali et al., 2016). In this review, therefore, we decided to focus on a narrow definition of *in vivo* or naïve BM-MSCs: the MSCs that form the part of HSC niche in BM. In this regard, CD146 and CD271 MSCs were found to localize in and close proximity to the HSC niche (Tormin et al., 2011). Tormin and his coworkers demonstrated the *in vivo* capacity of CD271+BM-MSCs to form the bone marrow stromal niche that also supports HSCs. (Tormin et al., 2011). The group further characterized the phenotype of both CD271+/CD146+ and CD271−/CD146+ BM-MSCs and demonstrated that while the CD146 expressing MSCs was localized in the perivascular niche; the CD271 MSCs were localized in the hypoxic niche. Additionally, we were also able to confirm the MSC phenotype of BM-derived CD271+/CD45- cells (Das et al., 2013) and their *in vivo* localization in the hypoxic niche (Garhyan et al., 2015).

Both these CD271+ and CD146+ MSCs could not be maintained in the *in vitro* serum rich media (Das et al., 2013), (Bakondi et al., 2009), thus, providing strong example of the failure of serum rich media to maintain naïve BM-MSC stemness state. Bakondi et al found that freshly sorted CD133+ BM cells containing CD271+ cells showed a marked loss of cell surface marker expression when cultured in serum rich media (Bakondi et al., 2009). Tormin et al reported similar findings of the loss of CD271 expression in the *in vitro* culture system using serum rich media (Tormin et al., 2011). Additionally, Li et al showed that CD271+ BM-MSCs, when cultured *in vitro* serum-rich media demonstrated the gain of CD140a, a cell surface marker that denotes the differentiation of the naïve CD271+ MSC phenotype (Li et al., 2016). Indeed, we confirmed that CD271+ BM-MSCs grown in serum-rich media showed rapid down regulation of CD271 gene, as well as the loss of CFU-F potential (Das et al., 2013). Thus, it appears that addition of serum rich media to culture naïve CD271+ MSCs probably induces differentiation to form primed MSCs.

Similarly, Stro-1+ naive BM-MSCs could not be maintained in serum rich media. Stromal precursor antigen-1 (Stro-1) is by far the earliest known cell surface marker used for the direct isolation of MSCs by immunomagnetic sorting of BM cells (Gronthos and Simmons, 1995). Stro-1, a 75kd endothelial antigen, is a cell membrane single pass type I protein that translocate from the endoplasmic reticulum to cell membrane in response to the depletion of intracellular calcium (Barkhordarian et al., 2011; Ning et al., 2011). Stro-1 is expressed on endothelial cells, as well as on mesenchymal cells. Stro-1 is used as a cell surface marker to isolate BM-MSCs in combination with negative selection against glycophorin-A (Simmons and Torok-Storb, 1991; Ning et al., 2011). Stro-1+ MSCs were found to possess the ability to differentiate to HSC-supporting fibroblasts, smooth muscle cells, adipocytes, osteoblasts, and chondrocytes (Dennis et al., 2002). High expression of Stro-1 in MSCs was found to be associated with differentiation of cells toward osteoprogenitor and pre-osteoblasts cells lineages (Byers et al., 1999; Gronthos et al., 1999). Further studies indicated that combined expression of Stro-1, and CD146 might indicate the osteogenic differentiation commitment of MSCs (Shi and Gronthos, 2003). Thus, Stro-1 expression might be important for the osteogenic stage of MSC differentiation, and could also be involved in HSC niche. However, when these sorted Stro-1+ cells were cultured in serum rich media, the Stro-1+ expressing cells could not be maintained and the CFU-F forming ability was decreased (Gronthos and Simmons, 1995).

The use of serum rich media to maintain naïve MSCs posed several difficulties, which led the investigators to look for different alternatives including the use of serum free media culture system. Several attempts were made to maintain the pure culture of Stro-1+ BM-MSCs in serum free media using growth factors. However, much success has not been achieved (Table 2). In 1995, Gronthos et al attempted to maintain flow cytometry sorted Stro-1 BM-MSCs in an *in vitro* serum-free alpha-MEM culture system with various growth factors and or L-ascorbate (Gronthos and Simmons, 1995). Stro-1+ BM-MSCs cultured in presence of growth factors indicated an increase in CFU-F potential. Whereas, loss of CFU-F generating capacity was observed in pure CD271+ BM-MSCs population when cultured in serum free culture supplemented with FGF-2 (Battula et al., 2009). Additionally, BM-MSCs indicated enhanced expression of additional cell surface markers, such as CD166 and CD318. Thus, it seems that culturing pure MSCs alone in serum free media may not be able to maintain the stemness of these cells.

Cell surface markers other than CD271, CD146, and Stro-1 are used for the direct isolation of naïve MSCs, and then subjected to *in vitro* culture mainly using serum rich media. These candidate cell surface markers include CD106, CD73, CD105, FZD9, SUSD2, LEPR, and CD90 (Gronthos et al., 2003; Aslan et al., 2006; Battula et al., 2007; Sacchetti et al., 2007; Veyrat-Masson et al., 2007; Sivasubramaniyan et al., 2013; Li et al., 2016). These results suggest the possibility of utilizing additional cell surface markers for the prospective isolation of naïve BM-MSCs, and tracking of these cells *in vivo* in the HSC niche. However, it is not yet clear about the fate of these cells when grown *in vitro* in serum rich or serum free media.

## Overcoming the challenge: taking a stemness-based approach

To improve our existing knowledge on the naïve state of BM-MSCs, it is important to pursue an alternative approach. We suggest that taking a stemness-based approach might help us to gain novel insights about how these naïve MSCs are maintained in their own niche. The concept of stemness in MSCs has been obscured by identification of heterogeneous primed MSC population having different degree or level of stemness (Billing et al., 2016). At present, stemness of MSCs is increasingly defined in terms of CFU-F yield, and the expression of ES cell related stemness markers, such as Oct-4, Nanog and Sox-2 by primed MSCs. Furthermore, global gene expression profiling and proteomic approaches have been utilized to identify set of genes and protein markers (including secretome) that could define the undifferentiated state of MSCs. This approach has been adopted to define the molecular signature of undifferentiated state of MSCs. However, enormous heterogeneity of primed MSCs (*in vitro* expanded MSCs in serum rich culture) make it almost impossible to perform comparative gene expression analysis to search for common MSC related gene signature from published data. Most importantly, it is not certain whether the stemness of primed MSCs is acquired or intrinsic to naïve MSCs. For example, the expression of ES related transcription factors Oct-4, Nanog and Sox-2 in primed MSCs could be acquisitonal. Indeed, we found that in naïve CD271+ BM-MSCs, Oct-4 was not expressed (Das et al., 2013).

An alternative approach is needed to define MSC stemness in terms of stem cell niche, and developmental ontogeny. Such an approach on stemness has been applied to HSCs to develop *in vitro* culture techniques that can mimic the *in vivo* niche (Schofield, 1983; Arai and Suda, 2008). In HSCs, stemness incorporates two attributes: self-renewal and undifferentiated state. The stemness state is maintained by interaction of these cells with their niche in a gradient manner, where some cells may be overly dependent than others. Therefore, stemness could be broadly divided into extrinsic (niche dependent) and intrinsic (niche independent or self-sufficient) stemness. The extrinsic stemness was previously referred as strong-niche type (Schofield, 1983; Laplane, 2015), whereas intrinsic stemness was described as weak-niche type, where stem cells are partially dependent on niche (Schofield, 1983; Arai and Suda, 2008). Previous findings suggest that highly undifferentiated stem cells might exhibit intrinsic or weak-niche type. For example, primitive CD133+ HSCs could be better maintained *in vitro* than CD34+ HSCs (Gallacher et al., 2000) suggesting a relatively higher degree of intrinsic stemness in the former cell type.

The undifferentiated state of stem cell is greatly influenced by the developmental ontogeny of stem cells. For example, CD133 is a marker expressed by ES cells and also primitive HSCs, neuronal stem cells and endothelial progenitor cells (Yin et al., 1997; Carpenter et al., 2004; Kania et al., 2005; Codega et al., 2014; Sekine et al., 2016) and therefore, CD133+ HSCs could be considered as more primitive than CD34+ HSCs. Likewise, naïve ES cells is ontogenically more primitive than primed ES cells, as the former is derived from inner cell mass, while the later is derived from epiblast. Naïve ES cells exhibit higher degree of intrinsic stemness than prime ES cells (Hanna et al., 2010), as the former exhibit self-sufficiency (Ying et al., 2008), an extreme form of intrinsic stemness that require no external support to maintain stemness (Ying et al., 2008; Das, 2014).

Thus, primitive stem cells might exhibit a higher degree of intrinsic stemness than lineage committed stem cells. In this perspective, stemness could have a relational component to developmental ontogeny, where, primitive stem cells may exhibit a higher degree of intrinsic stemness than lineage committed stem cells. We speculated that it might be easier for primitive stem cells to maintain naïve stemness state in the *in vitro* culture than lineage committed stem cells.

Therefore, we considered a niche and developmental ontogeny approach to develop *in vitro* culture of naïve BM-MSCs. Our challenge was that a developmental ontogeny based approach has not been actively discussed within the MSC research field. Reasons might include a lack of understanding on the embryological basis of MSC existence in BM and other tissues. In the literature, we found some discussion and speculation that MSCs might originate from the somatic lateral plate mesoderm (LPM), as the MSCs could differentiate into cell types that ontologically come from the somatic LPM (Sheng, 2015; Montali et al., 2016). Furthermore, recent studies indicate that MSCs could also arise from neural crest stem cells (Muller et al., 2008). In this context, CD271 is both a marker of neural crest stem cells as well as MSCs, and therefore, CD271+ MSCs might be ontogenically more primitive than CD146+ MSCs. Furthermore, some CD271+ MSCs are positive for CD133, a marker highly expressed in embryonic stem cells, primitive HSCs, and primitive neural stem cells (Yin et al., 1997; Carpenter et al., 2004; Kania et al., 2005; Codega et al., 2014). On the other hand, CD146+ and Stro-1+ are the markers of pericytes and endothelial cells respectively (Ning et al., 2011; Tormin et al., 2011) and could differentiate mainly to osteogenic progenitors (Shi and Gronthos, 2003). Thus, it could be presumed that CD271+/CD133+ MSCs could be more primitive than Stro-1+ MSCs in terms of their developmental origin.

If we now consider the idea that primitive stem cells may exhibit a higher degree of intrinsic stemness, we expect that CD271+ /CD133+ BM-MSCs could be maintained in the *in vitro* culture. However, it appears that high serum media led to differentiation of these cells (Bakondi et al., 2009), and serum-free culture without growth factor supplements could not maintain these cells *in vitro* (Battula et al., 2007; Das et al., 2013) indicating that these cells are not entirely niche- independent.

Therefore, we took a co-culture based approach of MSCs and HSCs to simulate *in vivo* microenvironment of the BM niche, where both these cell types reside in close proximity. Previous work on CD133+ BM cell *in vitro* culturing indicated that the culture of both CD45+ and CD45- cells in a serum free medium with growth factor supplements could maintain both the hematopoietic and mesenchymal population. Thus, Gallacher et al maintained CD133+ BM cells in a serum-free liquid suspension culture containing IMDM supplemented with 1% BSA, 5 ug/ml of human insulin, 100 ug/ml of human transferrin, 10 ug/ml of low-density lipoprotein, 10^∧^-4 M beta-mercaptoethanol and growth factors (SCF, TPO, FLt-3, G-CSF, IL-3, and IL-6). This *in vitro* culture medium was able to expand the HSCs by 2-4-fold after 9 days of culture (Gallacher et al., 2000). Gallacher et al. (2000) Interestingly, Baksh et al demonstrated that MSCs could be maintained in the co-culture system with hematopoietic cells (Baksh et al., 2003, 2005). Additionally, the group reported that CD123+/CD45- mesenchymal progenitor cells with osteogenic differentiation ability could be expanded and maintained *in vitro* for 3 weeks (Baksh et al., 2003, 2005). These results indicated that the co-culture probably mimics some degree of the *in vivo* environment of stem cell niche. We suggest that the serum free co-culture could be termed as the stemness based *in vitro* culture method.

We have adopted this stemness-based *in vitro* culture method to maintain CD271+ BM-MSCs (Das et al., 2013). Table 1. We used serum free plus supplements of SCF, TPO, and Flt-3. The other three growth factors used to culture CD133+ HSCs, IL-3/IL-6, and G-SSF were not used, as our primary goal was to maintain the undifferentiated naïve MSCs in their quiescent native state and avoid the rapid expansion that could lead to differentiation. In other words, we wanted to keep these naïve MSCs in their physiological quiescent state. In this manner, we were able to maintain the undifferentiated CD271+/CD45- cells for a 2-week period and avoided their rapid expansion. At the end of the 2 week period, we evaluated the phenotype of these cells and found that they maintained high CFU-F yield, high expression of CD271 and CD133 cell surface markers, and high expression of HIF-2α and HIF-1α, two transcription factors, that could be involved in the self-renewal of MSCs (Lin et al., 2006; Yun and Lin, 2014), Table 1. Importantly, CD271+ BM-MSCs showed 2-fold expansion and thus maintained a quiescent state. By adopting the co-culture method, we were able to isolate the naïve CD271+ BM-MSCs from healthy human donors by direct immunomagnetic sorting of CD271+/CD45- BM cells followed by their culture *in vitro* with CD45+ cells in a 1:1 ratio, where about 1% cells are CD34+ HSCs (Garhyan et al., 2015). In this manner, we could sustain the quiescent state of CD271+/CD133+ BM-MSCs for 2 weeks *in vitro* (Garhyan et al., 2015).

Our findings that HSC co-culture could maintain the undifferentiated state of CD271+ BM-MSCs was further confirmed by Li et al. (2014). The group reported that growing CD271+ BM cells with human umbilical cord blood CD34+ cells can preserve the CD271+ cell surface marker expression in serum-free medium for a week, whereas culturing these CD271+ cells in serum-rich media led to the loss of CD271 marker expression.

## Validation of the stemness-based approach to culture naïve BM-MSCs

We have done preliminary evaluation of the stemness state of naïve CD271+ BM-MSCs with an ongoing goal to develop uniform criteria to define the stemness of these cells. The undifferentiated state of naïve CD271+ BM-MSCs was validated by evaluation of various genes including CD133, CD105, CD73, and CD90 cell surface markers (Das et al., 2013). Further investigations are needed to validate this *in vitro* culture system. For example, lineage differentiation capacity of CD271+ BM-MSCs maintained *in vitro* need to be studied. Lineage differentiation is an important aspect of stemness. A rigorous clonal analysis based study demonstrated the hierarchical model of lineage differentiation in primed MSCs (Muraglia et al., 2000; Lee et al., 2010; Russell et al., 2010). Findings of these clonal studies support the notion that within the MSC population, a cell state having different level of stemness or lineage commitment might exist. Future clonal analysis using the flow cytometry sorted naïve CD271+ BM-MSCs (Figure 1) could provide valuable insight about the role of this receptor in self-renewal and multipotency of naïve MSCs. In this context, we should also consider the heterogeneity aspect of CD271+ population. Li et al performed microarray based gene expression analysis to reveal insights about the CD271+ vs. CD271– population. They reported that all known MSC markers, including CD105, CD140b, Leptin4, CD106 were highly expressed in CD271+ subset in comparison to the CD271- population. Again, the expression of CD140a was very low in CD271+ population in comparison to CD271- subset. In conclusion, they found that CD271+/CD140low/- subset is having high CFU-F formation capacity and CD271+/FGF3- indicating that CD271+ subset is a heterogeneous population (Li et al., 2014). Hence, lineage study is required to evaluate whether these subsets of CD271+ BM-MSCs exhibit different level of stemness or lineage commitment.

As stated above, there has been a tremendous interest for *ex vivo* isolation of MSCs and these efforts instead of bringing clarity have increased the prevailing confusion about the stemness of *in vivo* MSCs (Lv et al., 2014). Part of the problem is the lack of a definitive protocol that could be adopted by laboratories across the world. A serum free culture with defined growth factors could be adopted by laboratories across the world without major ambiguities associated with serum. Even then, we need a common set of assays to validate the stemness of *in vitro* cultured naïve MSCs so that our research could enhance the clarity in the field.

We suggest a common validation approach: (1) *In vitro* CFU-F assay, and *In vivo* self-renewal capacity in NOD/SCID mice (Haynesworth et al., 1992). (2) Maintenance of MSC ability to contribute to HSC niche *in vitro* and *in vivo* (Muguruma et al., 2006). (3) Maintenance of the *in vivo* homing capacity to BM (Muguruma et al., 2006) (4) Rigorous clonal analysis to demonstrate the hierarchical model of lineage differentiation (Muraglia et al., 2000; Lee et al., 2010; Russell et al., 2010). These are also important functional attributes of stemness. In addition to these attributes, there are two other attributes that need mentioning here. (5) The maintenance of healthy naïve MSCs i.e., absence of telomere damage/shortening, minimal accumulation of mutation, and or senescence. These indicators are essential to demonstrate that an *in vitro* serum free co-culture system could indeed maintain naïve MSCs in their *in vivo* stemness state without undergoing genotypic or phenotypic alteration. (6) Global gene expression profiling have been widely used to identify the transcriptional signature of specific stem cell genes and to gain insight into the signaling mechanism regulating their differentiation programs in ES cells including HSCs and MSCs. Similar gene expression analysis may be required to validate that CD271 BM-MSCs could maintain a stable gene expression profile during *in vitro* culture. Such a rigorous examination and validation is indeed required to find out whether the *in vitro* serum free co-culture system that we developed (Das et al., 2013) could maintain the naïve CD271+ MSCs *in vitro*. (7) Finally, validation of the developmental aspect of the cells. We suggest that in the future, we need to expand the MSC research to incorporate the developmental ontogeny of these cells, and how it relates to stemness. For example, the potential identification of stemness markers of primitive mesodermal stem cell state (Trombi et al., 2009) would help to define the naïve state of MSCs. This and other primitive MSCs might exhibit a higher level of stemness characterized by high CFU-Fs potential, multipotency (Lv et al., 2014), as well as high degree of intrinsic stemness that lead to self-sufficiency (Das, 2014).

The serum free method that we used could maintain the naïve MSC cells only for 2 weeks. Hence, further improvement in culture method is necessary to maintain naïve MSCs for a longer period of time. There are several methods being tried to maintain MSCs in their quiescent state that includes MSC culturing under hypoxia. We found that use of 1% oxygen to culture CD271+ BM-MSCs led to upregulation of HIF-1α, and downregulation of CD146 suggesting a potential alteration in stemness of these cells (Garhyan et al., 2015). Recently, Ciavarella et al demonstrated that a freeze-dried culture method could maintain stemness of the aorta derived MSCs (Ciavarella et al., 2015) suggesting that these cells could survive and maintain stemness in harsh conditions. Similar approach to culture naïve MSCs is not yet reported. Another culture method, the spheroid culture technique was also tried to maintain MSCs *in vitro* with limited success (Cesarz and Tamama, 2016). Furthermore, a hydrogel-based culture system was tried to grow both HSCs and MSCs to mimic *in vivo* microenvironment of BM (Sharma et al., 2012). Interestingly, the hydrogel culture is similar to methylcellulose media that we used to culture BM-MNC (Das et al., 2003). We found that methylcellulose media could expand MSCs (Das et al., 2003, 2008a). Therefore, we are currently considering the use of this methylcellulose based method for the *in vitro* maintenance and expansion of CD271+ BM-MSCs in a long-term co-culture with HSCs. As the field moves forward, we are expecting that innovative culture method will be developed for the long-term culture of naïve MSCs for therapeutic uses.

## Regulation of *In vitro* expanded naïve MSCs stemness: potential mechanisms

To take the emerging field of naïve MSCs forward, it is important to understand mechanisms that modulate the stemness of naïve MSCs. It is especially important in the context of regenerative medicine, because, modulating MSC stemness might enhance the regenerative potential of naïve MSCs.

We recognize that not many research studies have been undertaken to understand the mechanisms that could modulate the stemness of naïve MSCs. In this context, the *in vitro* serum free culture to maintain naïve CD271+ BM-MSCs could be useful to understand the regulation of stemness in many ways. We could exploit this culture system to study the potential cell surface marker based regulation of stemness. CD271 is a low-affinity nerve growth factor receptor (LNGFR), or nerve growth factor receptor (NGFR), and its belongs to the tumor necrosis factor superfamily (Thomson et al., 1988). Recently, it was found that CD271 is required for the stabilization of HIF-1 α transcription factor in mouse embryonic fibroblast and cerebellar granule neurons (Le Moan et al., 2011) but whether CD271 also stabilizes HIF-1α in naïve BM-MSCs and have a role in regulating stemness requires further study. CD133 or Prominin-1 is a 120 kDa pentaspan transmembrane glycoprotein cell surface marker having diverse functional roles in cellular metabolism, as well as stemness (Miraglia et al., 1997). CD133 is known to regulate HIF-1α, and epidermal growth factor receptor (EGFR) mediated proliferation in cancer stem cells (Soeda et al., 2009), although, its role in MSCs is not yet clear. Other MSC related markers, including Stro-1, CD146, and their potential role in modulating the stemness of MSCs also needs to be elaborated. These receptors might be involved in regulating both extrinsic and intrinsic stemness, and such a possibility could be studied by using the *in vitro* CD271+ BM_MSCs serum-free culture method.

Regulation of extrinsic stemness involves external growth factor or niche support. Among the growth factors, Fibroblast growth factor (FGF)-2 play an important role in maintaining the naïve state of human ES cells (Xu et al., 2005). Whether FGF-2 may play a similar role in maintaining the naïve state of human MSCs is not yet known. Numerous research reports indicated that FGF-2 could increase the proliferation and multilineage potential of primed MSCs (van den Bos et al., 1997; Tsutsumi et al., 2001; Solchaga et al., 2005; Sotiropoulou et al., 2006). Muraglia et al cultured BM mononuclear cells to obtain MSC clones. Out of 2 × 10^7^ mononuclear cells, they could find 256 clones. Culture of these clones in FGF-2 positive media led to two-fold increase in the percentage of multipotent progenitors. They concluded that FGF-2 might be involved in preventing differentiation of BM-MSCs (Muraglia et al., 2000). However, flow cytometry based phenotypic analysis of the mesenchymal clones was not performed. Similar clonal analysis of flow cytometry sorted naïve MSCs, and their culture in FGF-2 containing medium will provide direct evidence of the potential role of this growth factor in regulating stemness of naïve MSCs.

In addition to FGF2, other growth factors, including epidermal growth factor (EGF), hepatocyte growth factor (HGF), stem cell factor (SCF), and platelet-derived growth factor (PDGF) were implicated in the self-renewal of primed MSCs (Gronthos and Simmons, 1995; Tsutsumi et al., 2001; Eom et al., 2014; Li et al., 2014). Eom et al reported that expression of EGF, FGF-4 and HGF were down-regulated during serial passage of BM-MSCs (Eom et al., 2014). Other studies indicated the need for growth factors for MSC survival and to arrest senescence. The depletion of growth factors during serial passaging of MSCs was correlated with autophagy, senescence, and down-regulation of stemness through suppression of AKT and ERK signaling (Eom et al., 2014). These studies indicate that growth factors may modulate complex gene network architecture and transcriptional activities of MSCs to maintain their stemness. Therefore, transcriptional profiling and global gene expression profiling are required to delineate the pathways regulating the stemness architecture of naïve BM-MSCs (Song et al., 2006). Such an approach led to identification of numerous genes that might regulate self-renewal and multipotency of primed MSCs. These genes include actin filament-associated protein, frizzled 7, dickkopf 3, protein tyrosine phosphatase receptor F, and RAB3B (Song et al., 2006). Whether growth factors regulate these gene pathways to maintain naïve MSC stemness requires further research.

During past decade, various genomic arrays have highlighted putative molecular signatures that maintain the undifferentiated state in primed MSCs (Song et al., 2006). Indeed, Izadpanah et al demonstrated in a comparative study between humans and rhesus monkey that MSCs isolated from different species expressed embryonic stem cell markers, Oct-4, Rex-1, and Sox-2 for at least 10 passages (Izadpanah et al., 2006). Additionally, Song et al (Song et al., 2006) demonstrated the candidate genes that regulate MSC self-renewal and multipotency, including actin filament-associated protein, frizzled 7, dickkopf 3, protein tyrosine phosphatase receptor F, and RAB3B. However, all these investigations are primarily based on primed MSCs that are cultured and maintained in the *in vitro* conditions, which need to be validated by performing appropriate assays if they have a role in naïve MSCs as well.

In contrast to regulation of extrinsic stemness, the regulation of intrinsic stemness might involve the aspect of self-sufficiency, i.e. the ability of stem cells to maintain their stemness without any external growth factor or niche support (Das, 2014). Thus, self-sufficiency, denoted on the extreme right of the stemness spectrum (Figure 3), increases as the degree of intrinsic stemness increases. The self-sufficiency aspect of stemness has been studied in naïve ES cells, where several autocrine signaling mechanisms operate that not only prevent differentiation but also enhance the autocrine production of various growth factors (Ying et al., 2008; Hanna et al., 2010). Our own study in ES cells indicated that self-sufficiency in ES cells was associated to the increase level of FGF-2 (Das et al., 2012). These insights on the self-sufficiency aspect of ES cell stemness could be applied to study the self-sufficiency aspect of intrinsic stemness in naïve MSCs. We speculate that autocrine stemness pathway, such as VEGFR1/VEGF/HIF-1alhpa that maintain intrinsic stemness in cancer stem cells (Das, 2006; Tsuchida et al., 2008) might also play a role in the regulation of the intrinsic stemness in naïve MSCs.

The study on the self-sufficiency aspect of intrinsic stemness of MSCs might also incorporate the view of developmental ontogeny and associated signaling mechanisms, such as Wnt/beta-catenin and Notch signaling pathway. Such an approach will provide further evidence to the speculation that stemness is relational to developmental ontogeny, where, primitive stem cells exhibit a higher degree of intrinsic stemness than lineage committed stem cells.

## Modulation of stemness by hypoxic niche in BM

Hypoxia plays an important role during embryonic development (Simon and Keith, 2008), and also in the maintenance of quiescent HSCs in their BM niche (Zhang and Sadek, 2014). Similar role of hypoxia in maintaining naïve MSC stemness is not yet clearly known. Research on primed MSCs indicates that hypoxic microenvironment (usually 2–9% O_2_) could maintain primed MSC *in vitro*, enhances proliferative capacity and minimizing spontaneous differentiation (Simon and Keith, 2008). Fehrer et al. demonstrated that BM-MSCs cultured in 3% O_2_ concentration showed significantly increased *in vitro* proliferative lifespan before reaching senescence in comparison to MSCs cultured in ambient O_2_ conditions (Fehrer et al., 2007). Interestingly, the proliferative capacity of MSCs was found to be reduced significantly in 1% or less O_2_ concentration (Holzwarth et al., 2010) suggesting that very low oxygen tension may reduce growth, and keep the cells in their quiescent state.

Hypoxia induces the activity of two transcription factors, HIF-1α and HIF-2α, which are known to modulate multiple cell functions, including embryonic development, angiogenesis, stem cell pluripotency, regulating signaling of multiple cascades, including the self-renewal of HSCs (Lin et al., 2008). However, the potential role of hypoxia and HIFs in the self-renewal of naïve BM-MSCs is not yet clearly known. In this context, we recently found that HIF-1α is highly expressed in the naïve CD271+ BM-MSCs directly isolated from human BM (Garhyan et al., 2015). We found that culturing CD271+ BM-MSCs in 1% Oxygen for a week led to phenotypic changes in naïve MSCs that include upregulation of HIF-1α, and downregulation of CD146. We also used 100 umol/L deferoxamine mesylate, a hypoxia mimicking agent to culture the naïve MSCs and found similar results of HIF-1α induction and CD146 reduction (Garhyan et al., 2015). This result is expected as CD271+ BM-MSC resides in their hypoxic niche, whereas CD146+ BM-MSCs reside in the angiogenic niche. It could be speculated that CD271 receptor might have some functional role in the hypoxic localization of the BM-MSCs. Interestingly, CD271 could modulate HIF-1α in fibroblast (Le Moan et al., 2011) although such a possibility in BM-MSCs has not yet been studied. Further, detailed investigations are indeed required to understand the role of CD271 marker in the hypoxic localization of naïve MSCs, and modulation of stemness.

## Modulation of stemness by hypoxia/oxidative stress: insight to altruistic stemness

While hypoxia could aid to maintaining stemness, hypoxia/reperfusion or oxidative stress could act as a powerful threat against stemness. Hence, it is expected that oxidative stress could affect MSCs stemness, and therefore, reduce their regenerative capacity. In the contrary, MSCs has been found to contribute in the regeneration of tissues undergoing oxidative stress. In animal models of reperfusion, such as myocardial infarction, MSCs were found to home and contribute to repair/regeneration by secreting anti-oxidants and angiogenic factors (Prockop, 2017). However, it is not yet clear, how MSCs could maintain stemness in the microenvironment of oxidative stress. DNA damage associated with oxidative stress could activate p53 leading to differentiation of stem cells, including MSCs. We addressed this question in context of the ES cell model of oxidative stress, and uncovered the mechanism of altruistic stemness, as discussed below.

We speculated that stem cells might have evolved specific mechanisms to enhance stemness in sites of oxidative stress to become niche independent and niche-modulatory (altruistic) to serve their cyto-protective or altruistic purposes (Das et al., 2012; Das, 2014). We studied this possibility in an *in vitro* hypoxia/oxidative model of human ES cells. Briefly, we demonstrated in our previous work that a rare fraction of ES cells, the ABCG2+/SSEA3+ cells exhibited an enhanced state of stemness characterized by self-sufficiency and transient suppression of p53 transcription factor, when exposed to hypoxia/oxidative stress (Das et al., 2012). Importantly, this cell sub-fraction secreted anti-oxidants like glutathione that exerted cytoprotection to the rest of the ES cell community exposed to hypoxia/oxidative stress (Das, 2014). However, with the return of p53 to basal levels, these rare ES cells underwent spontaneous apoptosis, exhibiting altruistic behavior i.e. sacrificing its own fitness to enhance the fitness of the rest of the ES cell population. These ABCG2+/SSEA3+ altruistic stem cells (ASCs) not only exhibited an intrinsic stemness state (Figure 3) characterized by self-sufficiency, but also exhibited secretory and niche modulatory activity to execute stem cell altruism (Das, 2014). Thus, intrinsic stemness incorporates altruistic stemness (Figure 3), a type of stemness that is activated in response to micro-environmental stress. Such an idea of stemness, where an intrinsic property of a stem cell is emerged only in the right microenvironment has been considered as dispositional stemness (Clevers, 2016; Laplane, 2016). Importantly, we found that ASCs are niche modulating as the conditioned media of these cells could modulate HSC and MSC niche in mice treated with cisplatin (Das et al., 2012). Our findings suggest that the functional studies of intrinsic stemness should incorporate niche modulating altruistic behavior, i.e. the ability of stem cells to be not only niche independent but also niche modulating. This idea of altruistic stemness has tremendous implication in stem cell based regenerative medicine (Das, 2014).

Notably, such an idea of altruistic stemness might have relevance in understanding how MSCs might defend its BM niche during oxidative stress. It is possible that during oxidative stress in BM, some of the MSCs might reprogram to ASC phenotype to protect the stem cell niche. These ASCs would exhibit high expression of ES cell related transcription factors. In a mouse model of cisplatin-induced BM oxidative stress, we noted the marked appearance of Oct-4 expressing MSCs (Das et al., 2008a), although similar experiments are not yet conducted for human CD271+ MSCs. Interestingly, high Oct-4, Nanog and Sox-2 expressing MSC like cells were reported in human bone marrow, the so-called multilineage- differentiating stress-enduring cells (MUSE) cells (Katagiri et al., 2016). Future studies are required to investigate the stress-induced reprograming of naïve MSCs to ASC or MUSE like phenotype and their role in BM niche protection during oxidative stress.

Furthermore, the application of ASC idea might help us to re-interpret the conflicting data on the expression of ES cell related stemness factors in MSCs. There is an increasing tendency to incorporate the expression of ES cell related stemness factors to describe the stemness of MSC (Kolf et al., 2007). Pierantozzi et al critically evaluated the expression of these stemness factors and found that NANOG, but not OCT-4 and SOX-2, was expressed in primed human BM-MSCs. Interestingly, NANOG was not expressed in freshly isolated MSCs, but was detected only after the *in vitro* culture of hMSCs (Pierantozzi et al., 2011), whereas Riekstina et al found the expression of Oct4, and Nanog, but not SOX2 in primed human BM-MSCs (Riekstina et al., 2009). ES cell factors expressing were also detected in MSCs obtained from rhesus monkey subjected to serial passaging in the *in vitro* culture (Izadpanah et al., 2006). It is possible that stress-induced altruistic stemness might underlie the phenotypic observation of MUSE cells. It is possible that expression of these genes in primed MSCs might indicate some aspect of altruistic stemness that emergences during *in vitro* culture mediated oxidative stress. Such a possibility could support the notion that the *in vitro* culture induced oxidative stress might induce the niche modulatory or altruistic aspect of stemness in some of the MSCs. Future studies are thus required to find out if altruistic stemness is involved in regenerative potential of primed MSCs.

Unlike stem cell altruism, stem cell competition is involved in eliminating other stem cell clones to occupy the niche. This phenomenon is well studied by performing the HSC re-population assay in mice (Stine and Matunis, 2013). In this competitive repopulation assay, HSCs differing in stemness (mutation induced alteration in stemness) are transplanted to mice by intravenous injection, and the homing and self-renewal of HSCs are compared (Stine and Matunis, 2013). In this manner, it was found that having a low p53 stemness state increases the competitiveness of HSCs to occupy niches (Bondar and Medzhitov, 2010). This is interesting, because, one of the main features of altruistic stemness is a state of low p3 (Das, 2014). Hence, it could be speculated that competing stem cells might use the altruistic defense mechanism to occupy niches. This idea has relevance in the homing and regenerative ability of transplanted MSCs. Although, stem cell competition is not yet studied in MSCs, we did study stem cell altruism of MSCs in the context of cancer cells/MSC interaction. We found that altruistic CD271+ MSCs characterized by low p53 state showed significantly higher homing capacity than naïve CD271+ MSCs exhibiting extrinsic stemness (Talukdar et al., under review) supporting our view that altruistic stemness might contribute to stem cell competition to occupy niches. Taken together, the *in vitro* stemness based assay of CD271+ MSCs might serve as an experimental tool to study MSC competition and altruism, and how stem cells could exploit these two evolutionary mechanisms to maintain stemness during stress.

## Modulation of MSC stemness by immune system

In addition to growth factors, hypoxia, and oxidative stress, immune system/inflammatory activity could also affect the stemness of MSCs (Kolf et al., 2007). The *in vitro* naïve CD271+ BM-MSCs assay could be a valuable tool to gain insight on this possibility.

**Figure 4:**
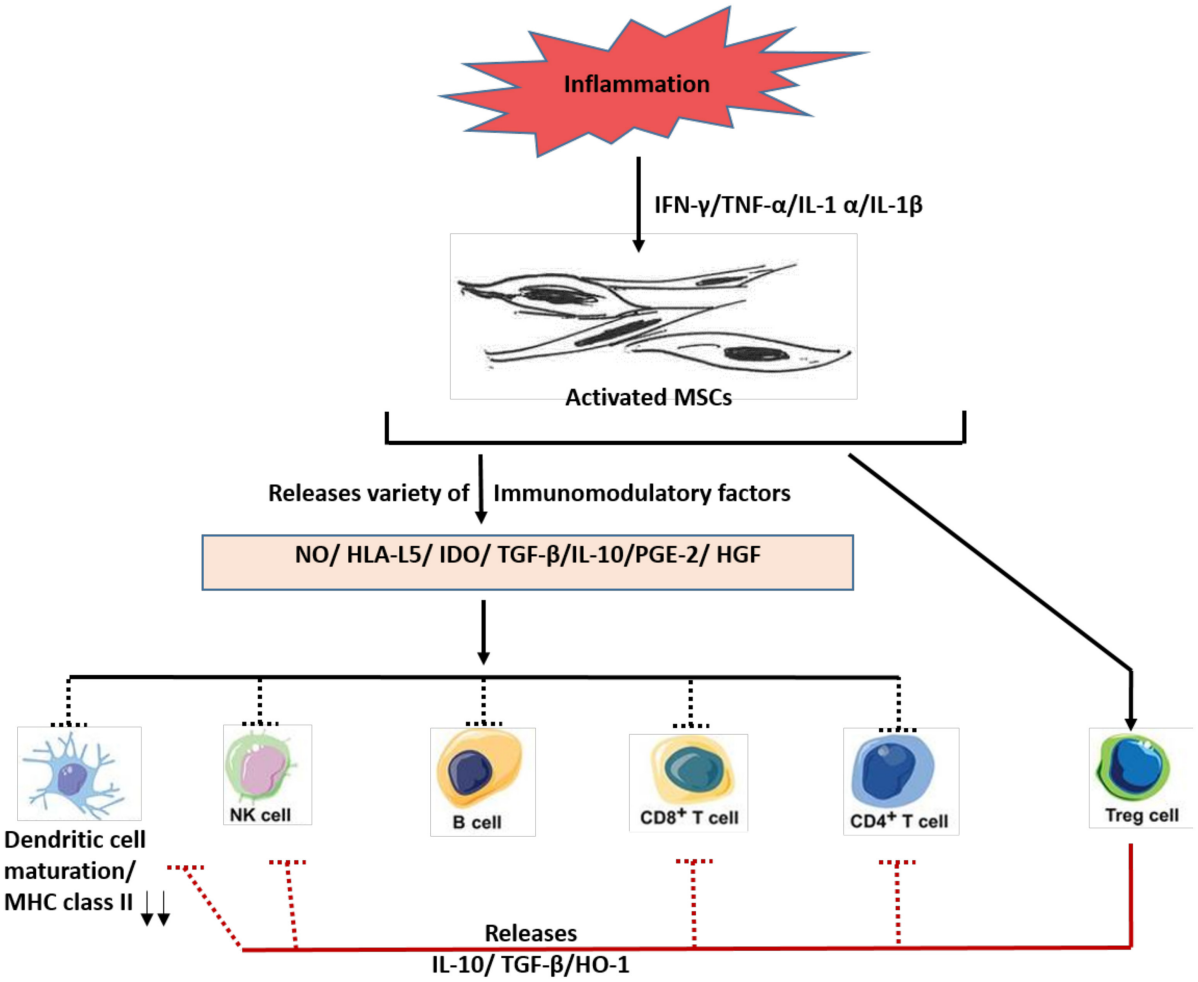
A schematic diagram depicting the immunosuppressive roles of MSCs. In response to inflammation, various molecules such as interferon–gamma (IFN-y), tumor necrosis factorα (TNF-α) and other cytokines are secreted, which activates MSCs. The activated MSCs then releases various immunomodulatory factors such as nitric oxide (NO), histocompatibility antigen L5 (HLA-L5), and other agents to mediate immunosuppressive actions. Much of the research work on immunosuppressive role of MSCs have been done using either primed human MSCs and or mouse MSCs. Future work is needed to confirm these findings using the naïve MSCs. IDO, indoleamine 2,3-dioxygenase; PGE-2, prostaglandin E2; HGF, hepatocyte growth factor; MHC, major histocompatibility complex; NK cells, natural killer cells; Treg cells, T regulatory cells; HO-1, heme oxygenase-1.

Immune system comprises of two major wings, innate and adaptive, which are known to modulate two broad immune reactions: immune activation and immune inhibition or suppression. For the tissue, regenerative process to be effective, a proper balance between both immune activation and suppression is required. Extensive interest in MSCs for their potential clinical use was fostered, mainly due to their immunosuppressive properties (Bartholomew et al., 2002). Indeed, several investigations demonstrated that primed MSCs releases different immune-modulators, such as nitric oxide (NO) (Sato et al., 2007), prostaglandin (PGE2) (Aggarwal and Pittenger, 2005), indoleamine 2, 3-dioxygenase (IDO) (Meisel et al., 2004), interleukin (IL)-6, IL-10, and HLA-G Figure 4. These secretory factors could modulate both the adaptive and innate immune responses by acting on T-cells, NK cells and other immune cells Figure 4. As shown in Figure 3, the capacity of MSCs to influence the fate of various immune cells is rather extensive. For instance, MSCs suppress T- lymphocyte activation and proliferation in response to antigens and non-specific mitogens (Di Nicola et al., 2002). MSCs could also reduce the secretion of interferon-gamma (potent inflammatory molecule) to enhance CD4+/CD25+ T regulatory cells, having potent immunosuppressive properties (Maccario et al., 2005). MSCs could also suppress the antigen presentation process by inhibition the maturation of resting natural killer (NK) cells (Spaggiari et al., 2008), and B cell proliferation (Corcione et al., 2006).

However, the demonstrated immunosuppressive effects of MSCs were performed by using primed MSCs. Whether naïve MSCs also exhibit immunosuppressive activity, and if so, what could be the physiological basis for such activity is not known. Nevertheless, based on the findings on primed MSCs, we could speculate that immune system might exploit naïve MSCs to maintain homeostasis between the two wings of immune reaction: pro and anti-inflammatory. Thus, in an inflammatory tissue having very high level of immune activation, the immune suppressive component would try to restore balance by recruiting MSCs. For this purpose, immune cells might secrete factors that could modulate the stemness of MSCs and hence its self-renewal. A recent study tends to support this hypothesis, albeit indirectly. Lee et al reported an autocrine role of PGE2 on MSCs. PGE2 is an immunosuppressive molecule. Binding of PGE2 to the E-Prostanoid (EP) 2 receptor on MSCs enhanced the self-renewal capacity of MSCs (Lee et al., 2016). This finding indicates that PGE2 could enhance the stemness of immunosuppressive MSCs. Interestingly, PGE2 is secreted by many inflammatory cells, including macrophages (Ikegami et al., 2001). Therefore, it could be speculated that immune cells could modulate the stemness of MSCs by secreting soluble factors. Such a role of immune cells might be important in the stem cell niche. One possibility is that PGE2 secreted by inflammatory cells present in stem cell niche might also modulate the stemness of MSCs present in the niche. In this manner, inflammation and immunity could exert influence on stem cell niche, and thus modulate extrinsic stemness (Niche dependent stemness). Hence, research on the potential role of the immune components in modulating the extrinsic stemness of MSCs needs attention.

In this context, it is also important to revisit some of the progresses made to understand the role of inflammatory molecules, such as Toll like receptors (TLRs) activators in modulating various properties of MSCs including immune modulatory activities.

Toll-like receptors (TLRs) are single membrane spanning receptors expressed in innate immune cells, including macrophages and dendritic cells (Akira et al., 2006; Satoh and Akira, 2016). TLRs belong to the pattern recognition receptor family that includes TLR1 to TLR10. These receptors recognize specially structured microbial molecules PAMPs (pathogen associated molecular pattern), and DAMPS (damage associated molecular patterns) and are expressed on human innate immune cells. Following ligand bindings by PAMPS or DAMPS, TLRs exert a variety of immune responses that include both pro and anti-inflammatory activities. In addition to immune cells, TLRs could also be expressed on stem and progenitor cells. Indeed, TLR2 and TLR4 were shown to be expressed by adult neural stem cells (Rolls et al., 2007).

The modulatory effect of TLRs on MSCs has been extensively reviewed by Delarosa et al. (2012). It appears that TLRs could regulate differentiation, proliferation as well as immune modulatory activities of primed MSCs, although the results are conflicting. Waterman and colleagues reported that specific TLRs activators, such as Lipopolysaccharide (LPS) or poly(I:C) mediate the polarization of MSCs toward either pro-inflammatory or anti- inflammatory phenotypes in the *in vitro* culture (Waterman et al., 2010). While TLR3 stimulation exerts immunosuppressive effects known as MSC 1 phenotype, activation of TLR4 provides a pro-inflammatory signature known as MSC2 phenotype. In contrast, in the *in vivo* animal models of sepsis or LPS-induced lung injury, only immunosuppressive activity was observed (Xu et al., 2007; Nemeth et al., 2009; Mei et al., 2010) suggesting that high level of LPS in mice did not polarize MSCs toward MSC2 or pro-inflammatory phenotype (Delarosa et al., 2012). Thus, it seems that *in vivo* or naïve MSCs could maintain their immunosuppressive phenotype despite bacterial sepsis mediated activation of TLRs. However, it is not yet clear whether TLRs could potentially modulate the stemness of MSCs to become pro-inflammatory.

## Modulation of MSC stemness by cancer and pathogens

Cancer cells and pathogens might modulate the stemness of MSCs for their benefits. We took advantage of the *in vitro* naïve CD271+ BM-MSCs assay to gain insight on this possibility, and our ongoing research in this area is reviewed below.

Cancer has been widely acknowledged as wounds that never heal, mainly because of its ability to remodel local tissue microenvironment and generate chronic inflammatory responses (Dvorak, 1986). Hence, it is not surprising that cancer cells might regulate the niche modulatory aspect of MSC stemness to remodel the microenvironment to facilitate its needs. Indeed, numerous studies demonstrated that cancer cells, including cancer stem cells (Visvader and Lindeman, 2012; Kreso and Dick, 2014) might recruit BM derived stem cells (mesenchymal, hematopoietic and endothelial) to sustain tumor growth and metastasis (Kaplan et al., 2005; Karnoub et al., 2007; Folkins et al., 2009). MSCs secrete several growth factors having pro-angiogenic and pro-tumorigenic activities. Vascular endothelial growth factor (VEGF), a highly potent pro-angiogenic factor secreted by MSCs (Schinkothe et al., 2008) might recruit MSCs to tumor sites and induce trans-differentiation of these cells to vascular cells. Indeed, in an orthotropic mouse model of pancreatic cancer, Beckermann et al reported that VEGF expression by tumor cells is directly correlated with rapid mobilization and recruitment of MSCs into neovascularization sites followed by their differentiation into vascular cells. Interestingly, VEGF secreted by MSCs induced sprouting of endothelial cells *in vitro* (Beckermann et al., 2008). In fact, various studies have demonstrated that MSCs might promote tumor angiogenesis via differentiation into endothelial-like cells or pericytes or by secretion of trophic factors and pro-angiogenic factors, cytokines, etc. (Rajantie et al., 2004; Bexell et al., 2009). However, several reports indicate that MSCs might also exert anti-tumor activity (Khakoo et al., 2006; Qiao et al., 2008), although the mechanism is not clearly known. These mixed results on pro and anti-tumor effects could be due to the use of primed MSCs in these investigations.

Our ongoing study indicates that naïve CD271+ BM-MSCs exhibit anti-tumor activity (Talukdar et al., 2016) on migratory cancer side population (SPm) cells, a type of cancer stem cells having high metastatic potential (Das et al., 2008b). Our findings support previous results that MSCs might exert anti-tumor activity (Khakoo et al., 2006; Qiao et al., 2008). We then speculated that SPm cells might modulate stemness of CD271+ BM-MSCs to niche modulatory or ASC phenotype to facilitate tumor growth. Indeed, we found that the conditioned media of SPm cells could reprogram CD271+ BM-MSCs to ASC like phenotype. These reprogrammed CD271+ BM-MSCs not only exhibited self-sufficiency but also indicated high secretion of VEGF and Stromal derived factor (SDF-1α), another pro-tumorigenic factor (Talukdar et al., 2016; Talukdar et al., under review). These reprogrammed CD271+ BM-MSCs or ASCs exhibited high expression of stemness related factors, including Nanog, Sox-2, Oct-4, as well as SSEA3 and CD105. Importantly, CD271+ ASCs showed a low p53 protein level, and demonstrated competitiveness in homing to BM in a competitive re-population assay performed in NOD/SCID mice. The homing of CD271+ ASCs facilitated cancer cell growth in the BM (Talukdar et al., 2016; Talukdar et al., under review). We like to note that the low p53 state facilitates HSC competition to eliminate competitors in the BM niche (Bondar and Medzhitov, 2010). Whether the low p53 state may also have facilitated the homing of CD271+ ASC is now under investigation, and results will provide insight about whether similar to HSCs, naïve MSCs exhibit stem cell competition. Nevertheless, our results demonstrated that cancer might exploit the regenerative capacity of naïve MSCs, and might manipulate both stem cell altruism and competition to enhance tumorigenesis and metastasis.

Similar to cancer cells, pathogenic bacteria may also exploit the regenerative capacity of naïve MSCs for their own benefit. Pathogen might target stemness potential of MSCs to avoid direct anti-bacterial activity of these cells. The anti-bacterial activity of MSCs was initially studied using *Pseudomonas aeruginosa*, a pathogen that causes pneumonia. Bonfield et al reported that human primed MSCs can exhibit anti-microbial activity in a sub-lethal cystic fibrosis mouse model of chronic *Pseudomonas aeruginosa* infection by secreting antimicrobial peptide cathelicidin, hCAP-18/LL-37 (Bonfield et al., 2013; Sutton et al., 2016). However, these impressive antibacterial actions of BM-MSCs are studied using primed and not naïve MSCs. Using the *in vitro* naïve CD271+ BM-MSCs culture model, we found that M. tuberculosis, the causative agent of pulmonary tuberculosis, could hijack these stem cells as a protective niche to evade immune reactions. Importantly, we demonstrated that the undifferentiated stemness state of naïve CD271+ MSCs and not primed CD271+ MSCs could maintain the dormant Mycobacterium tuberculosis (Das et al., 2013). Thus, it appears that modulating stemness state of MSCs might benefit the pathogen survival. Furthermore, we noted that CD271+ MSCs that harbor dormant M. tuberculosis showed a competitive advantage to home to BM in an *in vivo* transplantation assay. When CD271+ BM-MSCs that harbor GFP-labeled M. tuberculosis were transplanted to congenic mice, the MSCs harboring virulent strain H37Rv, showed an increase homing to BM compared to MSCs that harbor the avirulent strain mutant 18b (Das et al., 2013). These findings led us to speculate that virulent M. tuberculosis strain might modulate the stem cell niche (Garhyan et al., 2015), and or increase the stem cell competition of the infected MSCs by altering stemness e.g. extrinsic to altruistic stemness. Pathogen probably could achieve this task by modulating TLRs system in naïve MSCs, which we are now actively investigating. Hence, the naïve MSCs/M. tuberculosis host pathogen interaction could be used to study how pathogen could regulate the stemness state of naïve MSCs for bacterial pathogenesis.

The M. Tuberculosis host pathogen interaction could also help us to explore defense mechanisms of stem cell niche. We expect that the defense mechanism will involve niche modulation to prevent pathogen infection/invasion. In fact, studies showed that HSCs and endothelial progenitor cells in BM niche modulate pathogen infection (Nombela-Arrieta and Isringhausen, 2016), an indication of a niche based defense mechanism against pathogens (Garhyan et al., 2015). We are now actively studying whether naïve BM-MSCs are involved in this defense mechanism against pathogens including M. tuberculosis by inducing the niche modulatory aspect of stemness.

## Conclusion

BM-MSCs have generated considerable interest in stem cell based clinical and regenerative therapeutics due to their promising healing potentials. However, the exact naïve phenotype of this important cell type is yet to be defined and established. The culture expanded MSCs have been demonstrated to differ considerably from their naïve MSC counterpart. Thus, there is an urgent need to develop unique *in vitro* culture methods to maintain and expand the naïve counterpart BM-MSCs. In this context, our group has reported the culture and maintenance method for naïve CD271+ BM-MSCs obtained from healthy adult human by adopting a stemness based culture method. In this method, we demonstrated the culture of MSCs with hematopoietic cell population derived from CD133+ BM cells. This review intends to elaborate on the emerging concept of MSC stemness based on the stem cell niche, and developmental ontogeny. Such an approach will not only help us to gain insight about the physiological role of MSCs in their niche, but also the potential exploitation of these cells by cancer and pathogens.

## Author contributions

BP and BD wrote and edited the manuscript. BP created the figures. All authors reviewed the manuscript.

### Conflict of interest statement

BD is the founding director of KaviKrishna USA Foundation, Lincoln, MA and KaviKrishna Foundation, Sualkuchi, Assam, India. These foundations are Not-for-Profit, Non-Governmental organization to promote research on stem cell altruism. The KaviKrishna Foundation, Assam manages the KaviKrishna laboratory in Guwahati Biotech Park, IIT-Guwahati, Assam, India. The other authors declare that the research was conducted in the absence of any commercial or financial relationships that could be construed as a potential conflict of interest.
